# Prostatic Utricle Cyst as the Most Likely Cause in a Case of Recurrent Episodes of Hematospermia

**DOI:** 10.1155/2017/7502878

**Published:** 2017-12-21

**Authors:** Grégoire Feutry, Thomas De Perrot, Gregory J. Wirth, Xavier Montet, Steve P. Martin

**Affiliations:** ^1^Radiology, Department of Imaging and Medical Information Sciences, Geneva University Hospitals, Geneva, Switzerland; ^2^Urology, Department of Surgery, Geneva University Hospitals, Geneva, Switzerland

## Abstract

Hematospermia is a clinical symptom that raises anxiety in patients and has various causes, benign and malignant. We report a case of hematospermia for which appropriate multidisciplinary expertise favored a conservative management of a benign prostatic cyst, namely, a prostatic utricle cyst. A cystic lesion found by transrectal ultrasound in the context of hematospermia related to masturbation in a young virgin male patient was investigated with a high-field magnetic resonance imaging (MRI) and an endorectal coil. The association of high-field MRI and endorectal coil leads to high quality images.

## 1. Introduction

Hematospermia or hemospermia, defined by the presence of blood in the semen, is a clinical symptom that raises anxiety in patients. Most of the time, hematospermia is idiopathic and self-resolving [1]. A single occurrence is regarded as benign. In the young population with low risk factors, reassurance is sufficient. For patients over 40 years of age, hematospermia requires further investigations. Causes of hematospermia include sexually transmitted diseases, trauma, prostatic inflammatory or infectious disease, malignant neoplasia, cystic dilatation of the utricle, and conditions affecting the testis or the seminal vesicles [2, 3]. The management of hematospermia is influenced by the patient's age, the frequency of the episodes, and the associated symptoms. After a thorough physical examination, further investigations can be considered and include urethrocystoscopy, transurethral seminal vesiculoscopy, transrectal ultrasound, and MRI study. The transrectal approach allows an ultrasonography-guided opacification and dye-injection study that permits classifying the different types of midline cysts [4]. However, endorectal coil MRI is becoming the modality of choice for persistent symptomatology [5].

## 2. Case Report

A 15-year-old male originally from Guinea without sexual intercourse or history of perineal trauma complained about recurrent episodes of hematospermia when masturbating over the last 2 years. The patient did not report any other symptoms, especially macrohematuria or dysuria.

Clinical examination of the external genitalia and the abdomen was normal. Laboratory tests (blood count and urine test strip) did not reveal any abnormalities. Bacteriological tests were negative for* Chlamydia trachomatis*,* Neisseria gonorrhoeae*,* Mycoplasma*, and* Ureaplasma*. Virological and parasitic analyses were also negative.

**Figure 1:**
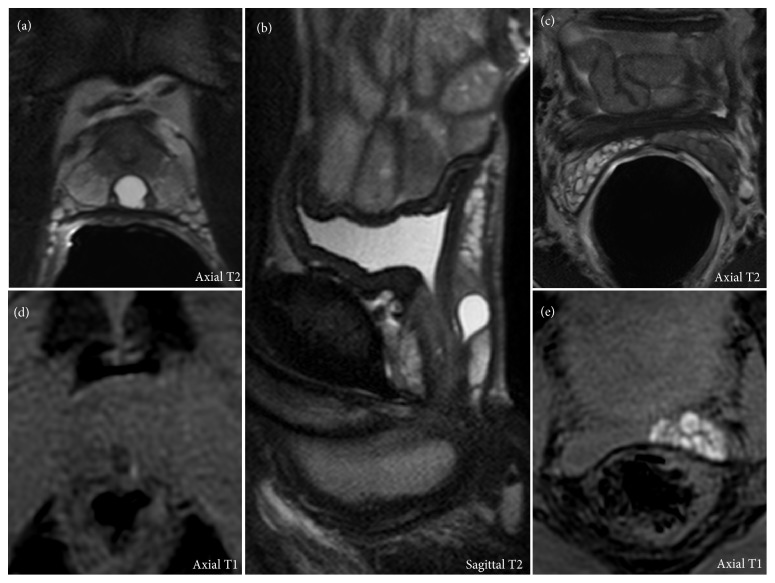
Summary sheet of 3-tesla MRI examination. Panels (a), (b), and (c) are acquired with an endorectal coil. Panels (d) and (e) are acquired without an endorectal coil and are the prebolus phase of the dynamic contrast-enhanced MR perfusion serving as T1-weighted images without gadolinium. Panels (a), (b), and (d) are centered on the cystic lesion. Panels (c) and (e) show stigmata of hematospermia in the left seminal vesicle. The orthogonal planes are specified in each panel.

Further investigations were pursued by transrectal ultrasound (TRUS) that showed an infracentimetric anechoic lesion located posteriorly to the prostatic urethra. This finding in association with the high frequency of occurrence of hematospermia justified the indication to a complementary magnetic resonance imaging (MRI). The exam was performed in a high-field 3-tesla unit with an endorectal coil. MRI confirmed the cystic nature of the prostatic lesion as a well-delimited median intraprostatic structure, hyperintense T2 and hypointense T1 (Figure 1, Panels (a), (b), and (d)). The pear-shaped intraprostatic cyst measured 7.5 × 8.5 mm in the axial plan and 12 mm in the cranial-caudal axis. The examination also depicted stigmata of hematospermia in the left ejaculatory duct and seminal vesicle such as low signal intensity on T2-weighted images (Panels (a) and (c)) and spontaneous high signal intensity on T1-weighted images (Panels (d) and (e)). A definite classification of this midline cyst would have required a minimally invasive approach with transrectal ultrasonography-guided opacification and dye-injection study [4], but the patient refused further investigations. Based on the young age of the patient and the size of the lesion, we concluded to a prostatic utricle cyst. The lesion being a solitary finding without significant clinical symptoms but occasional episodes of hematospermia, the patient was reassured and a conservative approach was chosen. After excluding all other causes, we retained this midline cyst as the most likely cause of hematospermia even in the absence of intralesional hemorrhage.

## 3. Discussion

Cysts of the lower male genitourinary tract are infrequent encounters. They are usually classified according to their topography, intraprostatic or extraprostatic. Extraprostatic cysts involve the seminal vesicles, the vas deferens, and the Cowper ducts. Those cystic entities are beyond the scope of our case and will not be further discussed. Intraprostatic cysts can be divided into three subgroups depending on the relation with the midline: median, paramedian, or lateral [6]. Paramedian cysts involve the ejaculatory ducts. Lateral cysts include retention cysts, cystic degeneration of benign prostatic hyperplasia, and prostatic abscess and can be associated with tumors.

Our case gives the opportunity to shortly review the median intraprostatic cysts that are characterized by the involvement of the midline posteriorly to the upper half of the prostatic urethra. A couple of decades ago, they were believed to be a single entity [7], but nowadays they are considered as two separate entities: prostatic utricle cysts and müllerian duct cysts.

Prostatic utricle cysts are embryologic remnants of the Müllerian duct system that communicate with the urethra. They affect predominantly males younger than 20 years. They can be associated with genitourinary malformations or infertility [8]. They might manifest with hematospermia as in our case, urinary tract infection, or incontinence. Utricle cysts are pear-shaped lesions usually smaller than 10 mm with a simple fluid signal on all modalities unless when complicated by infection or hemorrhage.

Müllerian duct cysts are focal failure of regression and saccular dilatation of the embryological müllerian ducts. They are mostly found in males of the age between 20 and 40 years. Contrary to the utricle cysts, they are generally asymptomatic but can lead to urinary tract retention or infection. Müllerian cysts are teardrop-shaped lesions, typically bigger than utricle cysts, and, unlike the latter, can extend above the base of the prostate and do not communicate freely with the prostatic urethra [6].

Treatment is recommended only for symptomatic lesions [9]. Procedure options are transperineal or transrectal aspiration, endoscopic section of the utricle meatus and transurethral marsupialization, and open surgery.

## 4. Conclusion

Correct diagnosis of a median intraprostatic cyst relies essentially on the anatomic topography, the size of the lesion, and the demographic data. When benign lesion is strongly suggested, a conservative management and firm reassurance are sufficient.
